# Prediction of complete regression in fertility-sparing patients with endometrial cancer and apical hyperplasia: the GLOBAL model in a large Chinese cohort

**DOI:** 10.1186/s12967-023-04671-w

**Published:** 2024-02-02

**Authors:** Xingchen Li, Yiqin Wang, Jiaqi Wang, Yuan Fan, Jianliu Wang

**Affiliations:** Department of Obstetrics and Gynecology, Peking University People’s Hospital, No. 11, Xizhimen South Street, Xicheng District, Beijing, 100044 China

**Keywords:** Fertility preservation treatment, LASSO, Atypical endometrial hyperplasia, Complete regression, GLOBAL model

## Abstract

**Background:**

Fertility preservation treatment is increasingly essential for patients with apical endometrial hyperplasia (AEH) and early endometrial cancer (EEC) worldwide. Complete regression (CR) is the main endpoint of this treatment. Accurately predicting CR and implementing appropriate interventions during treatment are crucial for these patients.

**Methods:**

We conducted a retrospective study involving 193 patients diagnosed with atypical AEH or EEC, enrolled from January 2012 to March 2022 at our center. We evaluated 24 clinical parameters as candidate predictors and employed LASSO regression to develop a prediction model for CR. Subsequently, a nomogram was constructed to predict CR after the treatment. We evaluated the performance of the nomogram using receiver operator characteristic (ROC) curve and decision curve analysis (DCA) to assess its predictive accuracy. Additionally, we employed cumulative curves to determine the CR rate among patients.

**Results:**

Out of the 193 patients, 173 achieved CR after undergoing fertility preservation treatment. We categorized features with similar properties and provided a list of formulas based on their coefficients. The final model, named GLOBAL (including basic information, characteristics, blood pressure, glucose metabolism, lipid metabolism, immunohistochemistry, histological type, and medication), comprised eight variables identified using LASSO regression. A nomogram incorporating these eight risk factors was developed to predict CR. The GLOBAL model exhibited an AUC of 0.907 (95% CI 0.828–0.969). Calibration plots demonstrated a favorable agreement between the predicted probability by the GLOBAL model and actual observations in the cohort. The cumulative curve analysis revealed varying cumulative CR rates among patients in the eight subgroups. Categorized analysis demonstrated significant diversity in the effects of the GLOBAL model on CR among patients with different total points (p < 0.05).

**Conclusion:**

We have developed and validated a model that significantly enhances the predictive accuracy of CR in AEH and EEC patients seeking fertility preservation treatment.

**Supplementary Information:**

The online version contains supplementary material available at 10.1186/s12967-023-04671-w.

## Introduction

Endometrial cancer (EC) ranks among the most life-threatening gynecologic malignancies in developed countries [1, 2]. In line with the full liberalization of the three-child policy, the Chinese government now encourages women to have children to promote sustainable economic and social development [3]. Nonetheless, EC remains the leading cause of female mortality worldwide, with 28% of diagnosed cases occurring in premenopausal women and 7.8% affecting individuals younger than 40 years [4, 5]. Traditionally, it is recommended that these women undergo a staging abdominal hysterectomy, bilateral salpingo-oophorectomy, and pelvic washing; with or without lymph node staging [6]. However, these therapeutic approaches are not feasible for selecting women with childbearing age who wish to preserve their fertility. As a result, fertility-sparing treatment (FST) with progestin has been employed in fertile women with early-stage EC and is increasingly adopted in the modern era [7]. Progestins have been widely and successfully used since the early 1960s in the treatment of advanced and metastatic endometrial cancer [8]. Conservative treatment options include oral high-dose progestins, such as medroxyprogesterone acetate (MPA) or megestrol acetate (MA) [9, 10]. Recently, the levonorgestrel-releasing intrauterine system has been used as an alternative to oral progestins to increase local progestin concentrations and reduce systemic side effects [11]. Complete regression (CR) is the most important endpoint and aims of patients who receive FST. Reports indicate a CR rate of approximately 76.2-81.4% for FST, with a pregnancy rate of 31.6% and a live birth rate of 28.0% [6], although the duration of FST varies across studies [12, 13]. In previous literature, it has been demonstrated that young women affected by EC and undergoing FST have achieved commendable CR rates and outstanding overall survival [14]. Despite several studies and meta-analyses, the early identification of CR risk factors is crucial for promoting enhanced multidisciplinary teamwork, increased surveillance, and improved planning [15]. Consequently, early recognition and intervention for CR play an increasingly vital role in treatment planning, EC prognosis, and measures to support pregnancy in patients undergoing FST [16].

At present, numerous studies have investigated the risk factors associated with CR. Previous investigations have indicated that women with obesity, insulin resistance (IR), or diabetes may have a higher risk of developing apical endometrial hyperplasia (AEH) or early endometrial cancer (EEC) [6]. Weight loss exceeding 10% has been shown to positively influence CR [17, 18]. Thus, for patients with metabolic syndrome, FST can still yield promising CR outcomes in AEH and EEC [19]. Increases of recurrent EC and AEH following primary conservative treatment, fertility-preserving re-treatment can also achieve favorable CR rates and successful childbirth [20]. Furthermore, other studies have identified prolonged treatment, combination therapy with metformin, and polycystic ovary syndrome (PCOS) as risk factors for CR [21–23]. Preliminary investigations suggest that metformin may serve as a beneficial adjunctive therapy with a synergistic effect when used alongside progestin treatment for AEH or EEC [24]. In our previous study, we observed a significant association between metabolic syndrome, insulin resistance, recurrence, and CR time [25]. Metabolic syndromes are found to play an essential part in the progression of EC, including metabolic risk score [26]. Despite the substantial impact and remarkable predictive accuracy of present studies on CR, no model has been developed that integrates a comprehensive array of influencing factors as many as ours to predict CR (including 24 clinicopathological parameters). Furthermore, our study represents the pioneering effort in consolidating numerous factors within a singular model to forecast CR. Notably, this model adeptly elucidates the individual contributions of each influencing factor to the overall CR prediction. Establishing a comprehensive prediction model for CR is not only beneficial for FST but also allows for the intervention of relevant risk factors during the treatment process to improve the CR rate.

In this study, we conducted a retrospective analysis of patients who underwent fertility-preserving treatment at our center and examined the risk factors associated with CR in the AEH and EEC cohort. We further integrated the clinicopathological characteristics of the patients and categorized them into different groups. Finally, we developed and validated a predictive model for early identification of CR. Our objective was to identify predictors and estimate the prognostic accuracy of CR in AEH and EEC.

## Methods

### Study design and participants

We conducted a retrospective investigation of patients who underwent fertility-sparing treatment at Peking University People’s Hospital (PKUPH) from January 2012 to March 2022. Informed consent was obtained from all patients for the utilization of their personal information in health research. We first deleted patients with significant missing data, which refers to following-up data, such as CR time. We tried to minimize missing data as much as possible and conduct long-term surveillance for patients. Additional follow-up channels would be added, such as contacting the patient’s family or search the patients’ healthy record in our center to obtain follow-up outcomes. Multivariate multiple imputation with chained equations was used to account for missing values on the enrolled parameters [27]. When the patients are under-treatment in our hospital, they are asked to sign an informed consent whether they are willing to donate their clinical data for scientific research. Afterwards, these patients are informed the advantages and disadvantages of the fertility preservation. The Ethics Committees of PKUPH approved this retrospective study (Approval No. 2022PHB379-001). We included patients who met the following criteria: women aged 18–40 years with a confirmed histological diagnosis of AEH or early EC, who desired FST and were treated with progesterone, and exhibited no signs of myometrial invasion on magnetic resonance imaging. Diagnostic biopsy for AEH or EC was performed using dilatation and curettage (D&C). Exclusion criteria encompassed ongoing medroxyprogesterone acetate (MPA) treatment, suspected advanced EC, severe medical conditions, and loss of medical records. The research group for this retrospective study we conducted was the fertility preserving treatment patient with complete regression group, while the control group was the non-CR group.

### Patient evaluation

We collected clinicopathological and follow-up data. Demographic and clinical information was extracted from medical records, including age at diagnosis, body mass index (BMI), gestation (G), parity (P), systolic blood pressure (SBP), diastolic blood pressure (DBP), pulse pressure (PP), fasting blood glucose (FBG), serum insulin, triglyceride (TG), high-density lipoprotein (HDL), cholesterol, low-density lipoprotein (LDL), CA125, menstrual regularity, gestation, parity, PCOS, thyroid disease, family history, hypertension, diabetes mellitus (DM), metformin use, serum insulin, histological types, treatment strategy, and immunohistochemistry (IHC) markers including ER, PR, Ki-67, and p53. In qualitative judgment, we classify positive staining of these markers as positive cases, regardless of their proportion. Gestation and parity included all of pregnancies consisting of the prior pregnancies. These parameters are chosen according to metabolic factors and related reference [28–30]. PCOS was diagnosed according to the Rotterdam 2003 criteria. We calculated the BMI and the homeostasis model assessment-insulin resistance (HOMA-IR) index [31]. The HOMA-IR value [FBG (mmol/L) × FINS (µU/mL)/22.5] was employed to assess IR status, with a HOMA-IR ≥ 2.95 indicating IR [32]. BMI was categorized as normal (BMI < 25 kg/m²) or overweight (BMI ≥ 25 kg/m²). Any missing data were obtained from primary care clinicians.

### Conservative treatment and evaluation of response to progestin treatment

Upon completion of a comprehensive evaluation and the determination by the multidisciplinary team that the patient was suitable for treatment, FST was initiated. Response to treatment was assessed histologically using specimens obtained during each hysteroscopy. CR was defined as the absence of hyperplasia or cancerous lesions. Once CR was achieved, the same treatment regimen was continued for an additional 2–3 months for consolidation. A hysteroscopy was performed 3 months after the initial CR to confirm remission. The duration of therapy required to achieve CR was calculated from the initiation of treatment until the first pathological diagnosis of CR, provided that that no hyperplasia or cancerous lesions were detected in two consecutive hysteroscopic evaluations. After achieving remission, all patients underwent follow-up examinations every 3 months, including pelvic examinations, tumor marker assessments, transvaginal ultrasound imaging studies, and histological evaluations through office-based endometrial biopsies.

### Construction and validation of the predictive model

We considered 24 independent candidate variables to predict the probability of CR. Initially, we conducted univariate analysis to identify variables for each subgroup and assigned coefficients based on the B-value. A Cox regression model was used for univariate analysis of the relationship between covariates and the probability of CR in response to progestin treatment. The relationship between the 24 factors and the CR status was analyzed using univariate analysis. In the comparison of 24 parameters between CR and non-CR groups, continuous variables were tested with t test, and categorical variable were tested with Chi-squared test. Continuous variables were described using medians, ranges and interquartile ranges; categorical variables were described using frequencies and proportions. Time to CR was measured from the progestin start date. Patients without an event of interest by their last follow-up date were censored in both analyses. There were no competing risk events in either analysis. Then, risk scores were calculated for each subgroup based on the coefficient. We employed Univariate Cox analysis to examine the correlation between risk factors and CR outcome, determining the coefficient of each variable in the model. Subsequently, to assess the impacts of potential predictors, we conducted both multivariable and LASSO Cox regression analyses. LASSO is a regularization and descending dimension method which can be used in biomarker screening for survival analysis combined with the Cox model [33]. Variables that demonstrated an association with CR in the univariable analysis were subsequently incorporated into the LASSO regression analysis. Using the LASSO regression results, a predictive nomogram was developed. To ensure user-friendliness and intuitive interpretation, all β coefficients were standardized, setting the lowest coefficient value to one, resulting in risk scores close to integers. To evaluate the reliability and predictive performance of the nomogram, both the cumulative CR rate and the ROC curve were utilized. These assessments confirmed the stability and predictive functionality of the nomogram.

### Statistical analysis

Continuous variables are presented as mean ± standard deviation (SD), while categorical variables are reported as number (N) and percentage (%). Student’s *t*-test or the Mann-Whitney U test was used to compare values between two groups. The Chi-square test and Fisher’s exact test were employed to compare the distributions of categorical patient and tumor characteristics. Survival curves depicting CR based on selected characteristics were generated using the Kaplan-Meier estimator, and differences were assessed using the log-rank test. Univariate and multivariable Cox regression models were utilized to evaluate the relationship between covariates and CR in response to treatment. All statistical analyses were conducted using R version 3.4.3 (http://www.R-project.org, The R Foundation) and EmpowerStats (http://www.empowerstats.com, X&Y Solutions, Inc., Boston, MA). A two-sided significance level of *P* < 0.05 was considered statistically significant.

## Results

### Characteristics of enrolled patients

**Fig. 1:**
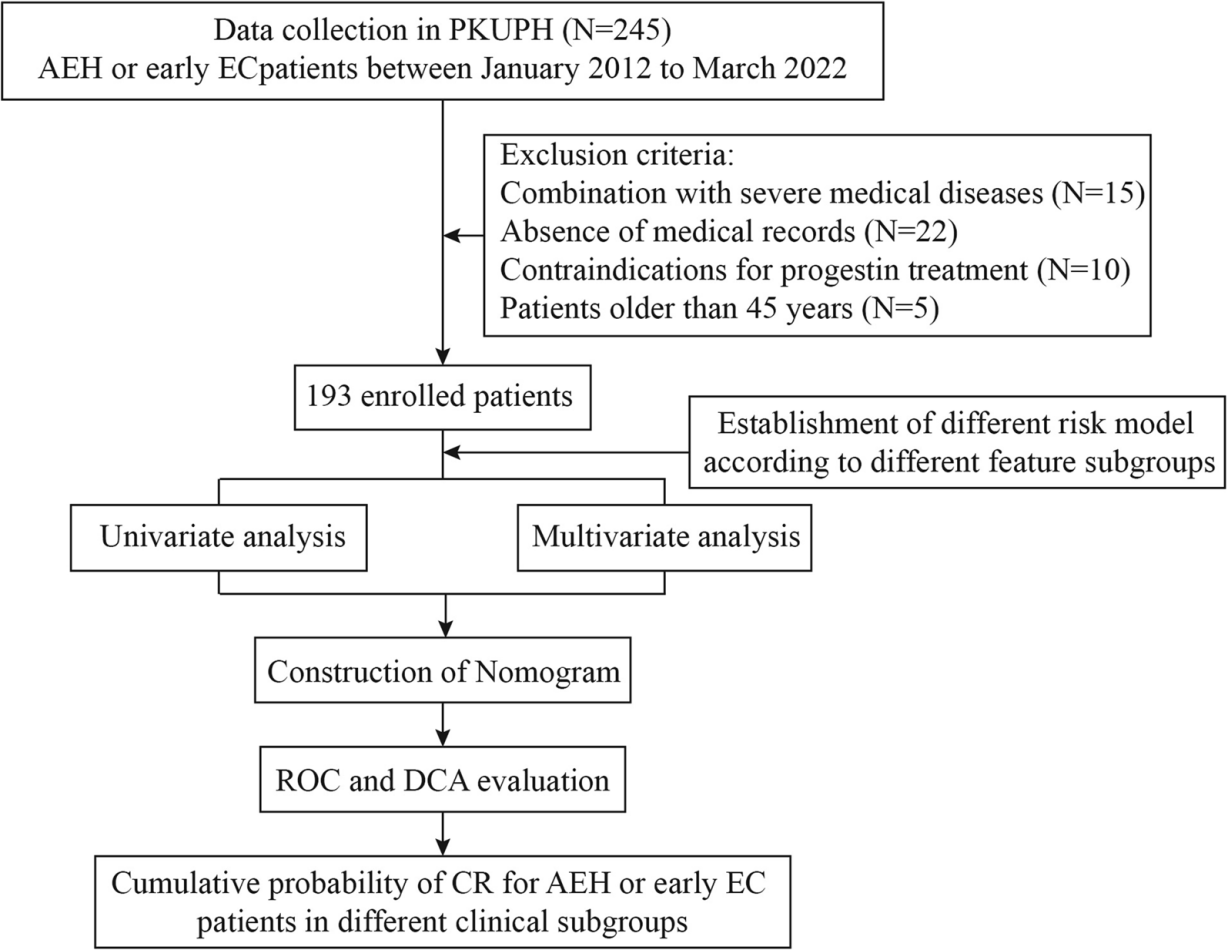
Flowchart of the study design

 The flow chart illustrating the patients’ enrollment in the study is presented in Fig. 1. A total of 245 patients underwent FSTs from January 2012 to March 2022. The final analysis included 193 patients, comprising 173 patients who achieved complete remission (CR group) and 20 patients in the control group who did not. The CR rate in our center was approximately 90%. A comparison of clinical variables between the two groups is provided in Table 1. *P* < 0.05 indicated that the distribution of the clinical parameters is significant different in the two groups. The median follow-up time was 10.15 months for the control group and 6.28 months for the CR group. The mean age of patients in the control group was 10.15 ± 7.55 years, while in the CR group, it was 6.28 ± 6.0 years. The me median BMI in the control and CR groups was 30.03 ± 4.33 kg/m^2^ and 26.87 ± 4.97 kg/m^2^, respectively. Serum insulin levels were significantly higher in the control group (29.79 ± 21.58 mmol/L). In the control group, 85% of the patients (n = 17) had irregular menstruation, while this proportion was 69.36% in the CR group. The number of patients diagnosed with PCOS was 9 (45%) in the control group and 58 (33.53%) in the CR group. Five patients (25.00%) were diagnosed with AEH in the control group, compared to 96 patients (55.49%) in the CR group. The remaining patients had EEC. There were notable differences between the two groups in terms of BMI, serum insulin, cholesterol, and LDL. Additionally, significant variations were observed in the distribution of patients in different parity, hypertension, histological type, maintenance treatment, estrogen receptor (ER) intensity, and progesterone receptor (PR) intensity (all *P* < 0.05). These findings indicate that the proportions and distributions differ in certain subgroups, and these characteristics may contribute to the achievement of complete remission in patients undergoing fertility preservation treatment for AEH and EEC.

**Table 1:** Baseline of enrolled patients with AEH and early EC in PKUPH

Variables	Control group	CR group	*P*-value*
	N = 20	N = 173	
Time to CR	10.15 ± 7.55	6.28 ± 6.01	0.011
Age (year)	33.60 ± 4.94	32.59 ± 5.02	0.222
BMI (kg/m^2^)	30.03 ± 4.33	26.87 ± 4.97	0.003
SBP (mmHg)	130.45 ± 17.31	123.05 ± 14.18	0.095
DBP (mmHg)	84.50 ± 13.56	79.27 ± 9.44	0.064
PP (mmHg)	45.95 ± 10.23	43.78 ± 9.95	0.322
FBG (mmol/L)	5.60 ± 3.04	5.41 ± 3.08	0.700
Serum insulin (mmol/L)	29.79 ± 21.58	17.00 ± 11.31	0.001
TG (mmol/L)	1.52 ± 0.84	1.57 ± 0.95	0.815
HDL (mmol/L)	1.12 ± 0.23	1.17 ± 0.33	0.599
Cholesterol (mmol/L)	5.18 ± 0.81	4.62 ± 0.85	0.005
LDL (mmol/L)	16.28 ± 57.13	2.92 ± 0.73	< 0.001
CA125 (IU/mL)	16.70 ± 8.82	19.54 ± 16.95	0.655
ER range( %)	0.73 ± 0.28	0.76 ± 0.21	0.970
PR range (%)	0.82 ± 0.18	0.75 ± 0.28	0.923
p53 range (%)	0.50 ± 0.48	0.30 ± 0.40	0.134
Ki-67 range (%)	0.27 ± 0.19	0.20 ± 0.13	0.184
Menstruation regularity			0.145
Yes	3 (15.00%)	53 (30.64%)	
No	17 (85.00%)	120 (69.36%)	
Gestation			0.358
No	17 (85.00%)	107 (61.85%)	
Yes	3 (15.00%)	66 (38.15%)	
Parity			0.034
No	19 (95.00%)	143 (82.66%)	
Yes	1 (5.00%)	30 (17.34%)	
PCOS			0.307
No	11 (55.00%)	115 (66.47%)	
Yes	9 (45.00%)	58 (33.53%)	
Thyroid disease			0.592
No	17 (85.00%)	154 (89.02%)	
Yes	3 (15.00%)	19 (10.98%)	
Family history			0.835
No	18 (90.00%)	153 (88.44%)	
Yes	2 (10.00%)	20 (11.56%)	
Hypertension			0.011
No	15 (75.00%)	160 (92.49%)	
Yes	5 (25.00%)	13 (7.51%)	
Diabetes			0.870
No	16 (80.00%)	141 (81.50%)	
Yes	4 (20.00%)	32 (18.50%)	
Metformin use			0.772
No	11 (55.00%)	101 (58.38%)	
Yes	9 (45.00%)	72 (41.62%)	
Medication use			0.109
Single	11 (55.00%)	125 (72.25%)	
Combined	9 (45.00%)	48 (27.75%)	
Maintenance treatment			0.030
No	8 (40.00%)	33 (19.08%)	
Yes	12 (60.00%)	140 (80.92%)	
Histological type			0.010
AEH	5 (25.00%)	96 (55.49%)	
Early EC	15 (75.00%)	77 (44.51%)	
ER intensity			0.014
+	6 (30.00%)	86 (58.11%)	
+ +	2 (10.00%)	21 (14.19%)	
+ + +	12 (60.00%)	41 (27.70%)	
PR intensity			0.005
Negative	0 (0.00%)	5 (3.40%)	
+	4 (20.00%)	72 (48.98%)	
+ +	2 (10.00%)	26 (17.69%)	
+ + +	14 (70.00%)	44 (29.93%)	
p53			0.408
Negative	4 (21.05%)	37 (30.33%)	
Positive	15 (78.95%)	85 (69.67%)	

AEH, apical endometrial hyperplasia; EC, endometrial cancer; CR, complete regression; BMI, body mass index; SBP, systolic blood pressure; DBP, diastolic blood pressure; PP, pulse pressure; FBG, fasting blood glucose; TG, Triglyceride; HDL, high-density lipoprotein; LDL, low-density lipoprotein; ER, estrogen receptor; PR, progestin receptor; PCOS, polycystic ovary syndrome

### Screening of risk formula in FST patients

**Fig. 2:**
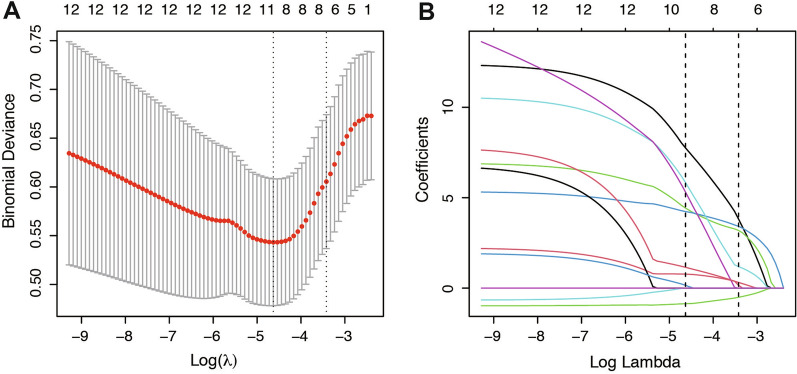
LASSO regression of risk factors from univariate analysis. **A** Ten-time cross-validation for tuning parameter selection in the least absolute shrinkage and selection operator (LASSO) model. **B** LASSO coefficient profiles of risk factors for FST patients

 To explore the risk factors associated with CR, we conducted univariate regression and multivariate Cox analyses. The patients’ clinical and pathological characteristics were divided into six groups based on their properties: basic information, characteristics, blood pressure, glucose metabolism, lipid metabolism, and immunohistochemistry. Univariate analyses were performed for each group, as well as well as other unclassified pathological features and CR. Table 2 presents the results, revealing significant correlations between CR and basic information (HR = 2.18, 95% CI 1.21–6.05; *P* = 0.014), characteristics (HR = 2.61, 95% CI 1.33–5.51; *P* = 0.006), blood pressure (HR = 2.35, 95% CI 1.33–5.71; *P* = 0.004), glucose metabolism (HR = 2.71, 95% CI 1.54–4.77; *P* = 0.001), lipid metabolism (HR = 0.76, 95% CI 0.47–1.21; *P* = 0.254), immunohistochemistry (HR = 2.68, 95% CI 1.47–4.90; *P* = 0.001), histological type (HR = 0.54, 95% CI 0.38–0.78; *P* = 0.001), and medication method (HR = 0.40, 95% CI 0.28–0.57; *P* < 0.001). Multivariate Cox analysis further identified basic information (HR = 2.35, 95% CI 1.05–5.27; *P* = 0.037), characteristics (HR = 3.78, 95% CI 2.02-279.71; *P* < 0.05), blood pressure (HR = 18.54, 95% CI 1.08-319.43; *P* < 0.05), glucose (HR = 12.54, 95% CI 1.23-127.98; *P* < 0.05), lipid (HR = 8.32, 95% CI 1.35–51.34; *P* < 0.05), immunohistochemistry (HR = 14.1, 95% CI 1.8-110.1; *P* < 0.05), histological type (HR = 0.63, 95% CI 0.43–0.93; *P* < 0.05), and medication method (HR = 0.46, 95% CI 0.31–0.68; *P* < 0.01) as independent risk factors. Subsequently, LASSO regression was employed for further risk factor selection (Fig. 2). The resulting signature, comprising the aforementioned eight risk factors, was constructed using their respective regression coefficients. FST patients’ CR score were calculated using the following formula: CR score = 3.21463 * BasicInform + 0.92370 * Characteristics + 2.46019 * GluMet + 0.98321 * BP + 1.03225 * IHC + 1.49781 * LipMet + 0.97184 * ComMed − 0.18491 * Histological type. These findings demonstrate that the identified eight factors remain significant in the LASSO analysis. Therefore, basic information, characteristics, blood pressure, glucose metabolism, lipid metabolism, immunohistochemistry, histological type, and medication method are valuable for predicting the prognosis of AEH and EEC patients undergoing FST. To enhance the predictive accuracy for CR, we developed the risk model named the GLOBAL model.

**Table 2:** Univariate and multivariate analyses of risk factors for CR in patients with AEH and early EC

Variables	Univariate analysis HR (95% CI)/*P*-value	Multivariate analysis HR (95% CI)/*P*-value
Basic information	2.18 (1.21, 6.05) 0.014	2.35 (1.05, 5.27) 0.037
Characteristics	2.61 (1.33, 5.51) 0.006	3.78 (2.02, 279.71) 0.011
Blood pressure	2.35 (1.33, 5.71) 0.004	18.54 (1.08, 319.43) 0.045
Glucose metabolism	2.71 (1.54, 4.77) 0.001	12.54 (1.23, 127.98) 0.037
Lipid metabolism	0.76 (0.47, 1.21) 0.254	8.32 (1.35, 51.34) 0.021
Immunohistochemistry	2.68 (1.47, 4.90) 0.001	2.194 (1.10, 4.35) 0.025
Histological type		
AEH	1.0	1.0
Early EC	0.54 (0.38, 0.78) 0.001	0.63 (0.43, 0.93) 0.019
Medication method		
Combined medicine	1.0	1.0
Single medicine	0.40 (0.28, 0.57) < 0.001	0.46 (0.31, 0.68) 0.001
Maintenance treatment		
No	1.0	
Yes	1.08 (0.74, 1.58) 0.701	

AEH, apical endometrial hyperplasia; EC, endometrial cancer; CR, complete regression; MRS, metabolic risk score; BMI, body mass index; PCOS, polycystic ovary syndrome

### Construction and validation of predictive nomogram for CR

**Fig. 3:**
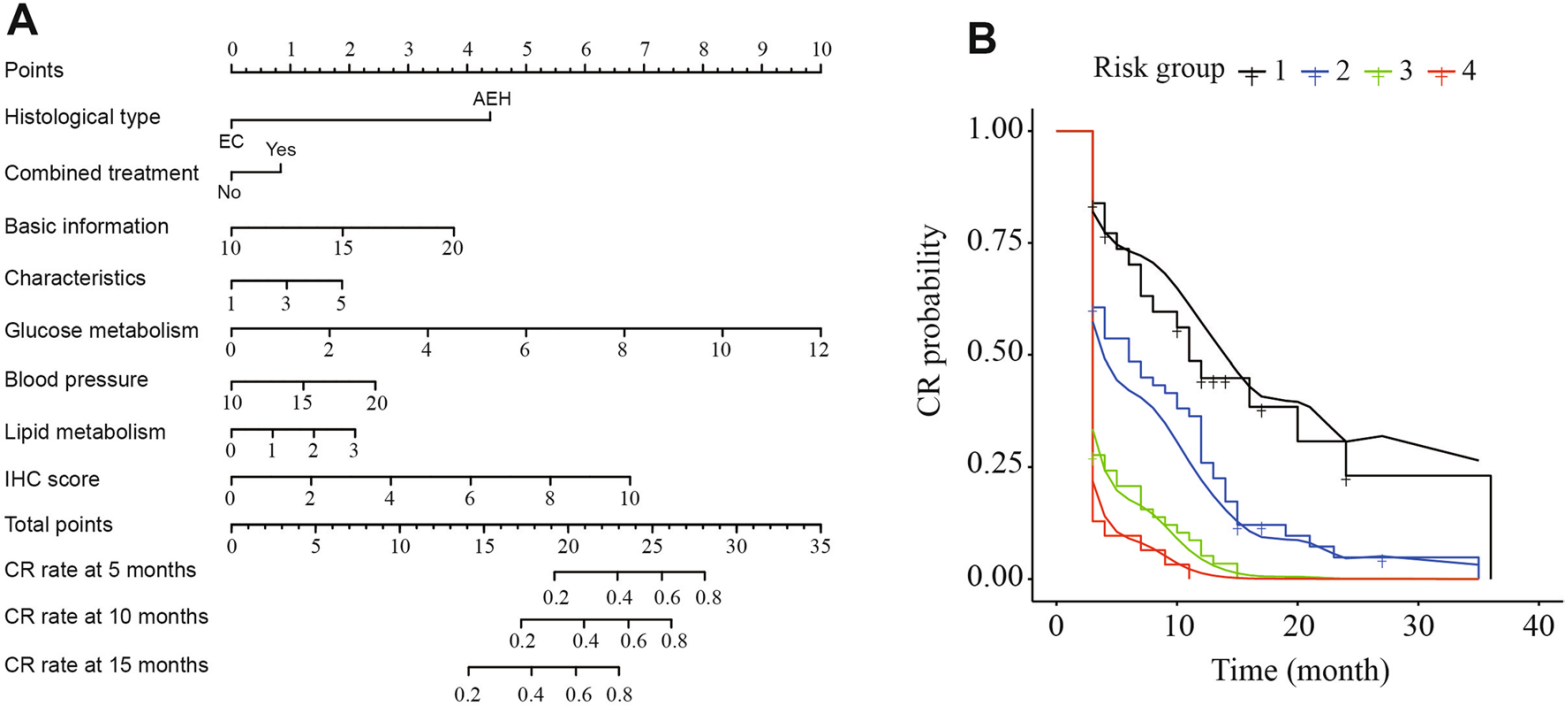
Construction and evaluation of the predictive nomogram. **A** Nomogram containing the risk factors to predict the CR rate in patients with FST. **B** CR rate of four risk groups categorized by different CR score

Using the eight risk factors identified from LASSO regression, we constructed a predictive nomogram to assess the probability of achieving CR in patients undergoing FST. Each variable was assigned a corresponding point, and the total points indicated the probability of CR. The nomogram is used to decide the probability of complete regression. In the nomogram, each factor is assigned a point. When the point of each factor are added up, the total points of a patient is obtained. In the nomogram, total points are corresponding with the 5-month, 10-month, and 15-month CR rate, as Fig. 3A shows. Therefore, the CR rate of patients at different times can be predicted based on each factor. The patients were then divided into four equally distributed risk groups. Survival probability analysis demonstrated favorable outcomes for all four risk groups (Fig. 3B). The C-index for predicting CR was 0.893, indicating a high level of accuracy. Calibration curves for the nomogram displayed good agreement between the predicted and observed outcomes (Additional file 1: Fig. S1). These results underscore the high predictive value and accuracy of the nomogram in forecasting CR during fertility preservation treatment.

### Evaluation of predictive accuracy for risk factors on CR

**Fig. 4:**
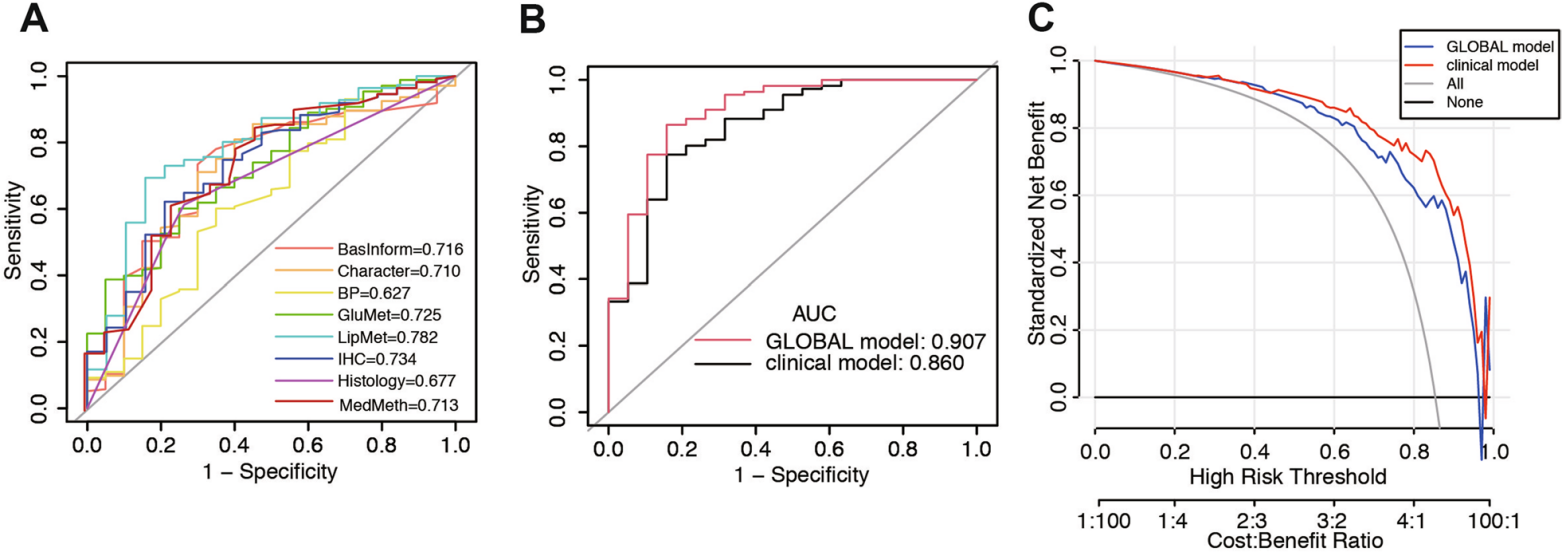
Evaluation of predictive accuracy with independent risk factors. **A** ROC of eight independent risk factors. **B** ROC curve of the clinical model and the GLOBAL model. **C** DCA curve for models with or without IHC.

To further assess the predictive value of the eight risk factors in AEH or EEC patients undergoing FST, we performed ROC curve analysis and DCA to evaluate their impact on CR. The ROC curve analysis using the eight risk factors revealed that most of the individual variables had an AUC greater than 0.7, including basic information (AUC = 0.716), characteristics (AUC = 0.710), glucose metabolism (AUC = 0.725), lipid metabolism (AUC = 0.782), immunohistochemistry (AUC = 0.734), and medication method (AUC = 0.713). The AUC was 0.627 and 0.677 for blood pressure and histological type, respectively (Fig. 4A). To further explore the significance of immunohistochemistry in the ROC model, we constructed a clinical model, and subsequently included metabolic-related features in the clinical + IHC model (GLOBAL model). As depicted in Fig. 4B, the ROC curve demonstrated an AUC of 0.860 for the clinical model, which increased to 0.907 after the addition of immunohistochemistry. This indicates a higher diagnostic accuracy for predicting CR in patients undergoing FST. Furthermore, DCA confirmed the predictive effectiveness of the model (Fig. 4C). The predicted probability thresholds were higher in the GLOBAL model, suggesting a positive net benefit for FST patients when immunohistochemistry was incorporated compared to the clinical model. Thus, these findings emphasize the significant role of immunohistochemistry in improving the predictive accuracy of CR following FST in patients with AEH and EEC.

### Cumulative curve of time to CR in different patients

**Fig. 5:**
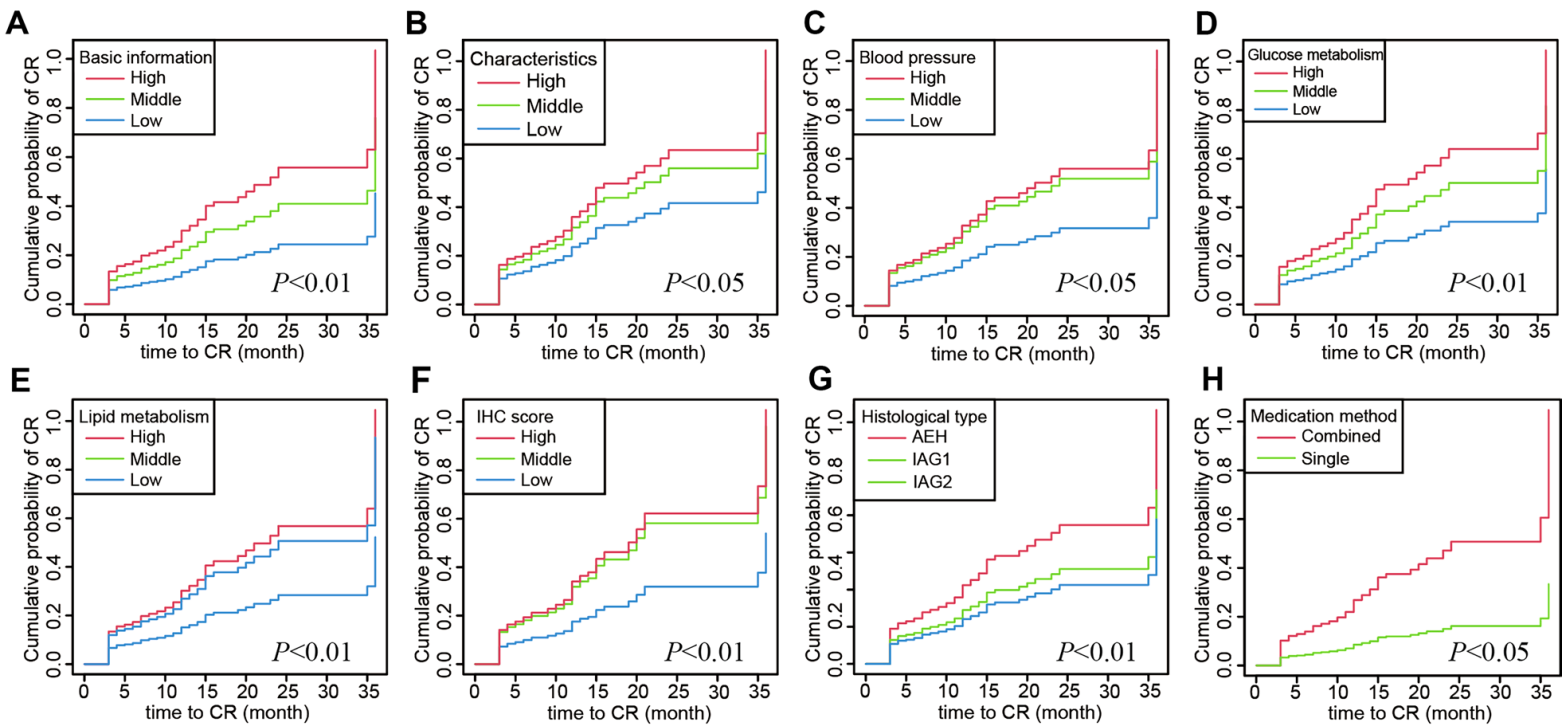
Cumulative probability of CR for 193 patients who underwent fertility-sparing treatment in subgroups with low-, moderate-, and high-score. **A** Basic information. **B** Characteristics. **C** Blood pressure. **D** Glucose metabolism. **E** Lipid metabolism. **F** IHC score. **G** Histological type. **H** Medication method

 To further illustrate the impact of the eight risk factors on CR, we generated a cumulative CR curve using stratified log-rank tests. The results revealed notable differences in the cumulative probability of CR among patients with different scores in basic information (Fig. 5A, P < 0.01), characteristics (Fig. 5B, P < 0.05), blood pressure (Fig. 5C, P < 0.05), glucose metabolism (Fig. 5D, P < 0.01), lipid metabolism (Fig. 5E, P < 0.01), immunohistochemistry score (Fig. 5F, P < 0.01), histological type (Fig. 5G, P < 0.01), and medication method (Fig. 5H, P < 0.05). These findings suggest that patients with lower scores, EC type, and single medication required a longer duration to achieve CR status.

## Discussion

This study represents the most comprehensive evaluation of risk factors associated with achieving CR in a large cohort of patients with AEH and EEC who underwent FST. The data analyzed in this retrospective study were derived from a single tertiary hospital in China. FST offers a promising option for young AEH and EEC patients who wish to preserve their fertility. By categorizing the clinical characteristics into different subgroups based on their nature, we identified eight significant risk factors for CR: basic information, characteristics, blood pressure, glucose metabolism, lipid metabolism, IHC, histological type, and medication use. These eight risk factors exhibit a high level of accuracy in predicting the prognosis of women who undergo FST for AEH or EEC. When combined, these factors yield an impressive AUC of 0.90, indicating excellent predictive accuracy. This study has important implications for clinical practice as it assists in prognosis estimation and enables physicians to make informed decisions regarding surveillance strategies for women with AEH or EEC. For instance, our findings highlight the impact of BMI on CR probability. Decreasing a patient’s BMI by 1 kg/m^2^ is associated with a 12.3% increase in the likelihood of achieving CR, while each additional year of age corresponds to a 4.7% decrease in CR probability. Consequently, we recommend weight loss efforts and prompt consideration of pregnancy for optimal outcomes. In summary, our study provides valuable guidance for managing FST inpatients with AEH or EEC, aiding in clinical decision-making and enhancing patient care.

Considering the recent increase in the in the incidence of EC among women of reproductive age and the favorable prognosis associated with this disease, it is crucial to offer effective FST options [34]. Fertility-sparing management, particularly in young women with presumed stage IA, grade 1, endometrioid carcinoma of the uterus who desire fertility preservation, has gained recognition and adoption [35]. However, the rates of achieving CR through FST vary across different studies. Progestin therapy has been widely reported as the primary approach for conservative management of EEC. The reported CR rates range from approximately 70–80% and exhibit variation among different cohorts. In our study, we reviewed data from 193 patients who underwent fertility-sparing treatment, including 173 patients who achieved CR and 20 patients in the control group. Thus, the CR rate in our cohort reached 89.6%. A systematic review of 25 studies encompassing 445 patients revealed an overall response rate of 82.4% to hormonal therapy [36]. Another study reported a pathological complete regression rate of 92.6% in patients with EEC and EAH treated with oral progestin, with a mean time to CR of 7.47 ± 2.91 months [37]. Similarly, an investigation of 179 patients demonstrated a CR rate of 94.4%, with 96.7% for AEH and 93.3% for EC [38]. Combination medication approaches have shown promising therapeutic outcomes. Zhou et al. reported an 88.2% CR rate in EC patients treated with a combination of gonadotropin-releasing hormone agonist (GnRH-a) therapy [39]. In our study, we observed that combined treatment significantly increased the probability of achieving CR, with a predictive accuracy of 0.713. It is worth noting that our center does not limit conservative treatment to a single regimen, and further research is necessary to ascertain whether this contributes to the risk of CR.

In the widely utilized risk classification for endometrial cancer, low-risk group patients are typically categorized as those with grade 1 or grade 2 endometrioid adenocarcinoma showing < 1/2 myometrial invasion, no cervical involvement, no lymph-vascular invasion, and no distant metastasis. However, these traditional classifications are insufficient to classify patients undergoing FST into high and low-risk groups. Thus, further exploration of new classifications for these patients is necessary. In a recent study proposing a molecular classifier for EC in fertility-sparing management, it was found that 15.8% of patients had mismatch repair (MMR) deficiency, and intact MMR function was identified as a predictive biomarker for improved hormone responsiveness [40]. Shen et al. conducted a retrospective analysis of patients with AEH or EEC using information from pathological reports. Molecular classification was performed using an 11-gene panel based on next-generation sequencing technology. Patients with copy number-high (CNH) and microsatellite instability-high (MSI-H) subtypes exhibited a worse prognosis compared to those with POLE-mutated and copy number-low (CNL) subtypes. Thus, molecular classification of AEH or EEC patients before progestin treatment could be feasible and may help identify patients at risk of progression [41]. A genome-wide DNA methylation analysis using pathological tissue specimens demonstrated that DNA methylation diagnostics based on the Youden index cutoff value for 6 out of 8 CpG sites (LPP, FOXO1, RNF4, EXOC6B, CCPG1, RREB1, and ZBTB38) could be applicable for risk estimation in FST patients aged 40 years or younger with EEC [42]. Another small study involving a 10-patient cohort revealed that levels of ER and progesterone receptor B (PRB) after treatment with a progestin-containing intrauterine device (IUD) were significantly higher in the “progression” group compared to the “no progression” group [43]. Additionally, a systematic review indicated that PR is a significant predictor of response in endometrial hyperplasia (EH) and EC treated with a levonorgestrel-intrauterine device, but not with oral progestins [44]. However, other studies did not report any significant associations [45]. The GOG211 trial found that only low pre-treatment levels of Ki-67 were predictive of histologic response, rather than ER or PR [46]. To enhance the accuracy of CR prediction, we classified patient characteristics and utilized the risk score of the classification to construct a nomogram. The prediction model we developed for CR achieved an AUC of 0.907, making it the most accurate predictive model for FST patients with AEH or EEC to our knowledge. We also found that menstrual regularity are positive related with CR rate. This may be because regular menstruation might partially indicate an aspect of ovarian function and can serve as a reminder for the cycle of hormone level. The stability of hormone levels in the body contributes to the rate of CR [47].

Apart from our study, other papers have also discussed the risk factors of FST for AEH and EEC. It has been observed that longer treatment duration is associated with higher rates of CR. Prolonged medical management may contribute to increased CR rates, as some initial non-responders eventually achieve CR at a later time point. For instance, the 12-month CR rate was 68.9%, but it reached 91.1% at 24 months [48]. The combination of a gonadotropin-releasing hormone agonist and aromatase inhibitor (AI) has shown promising long-term effects in young obese EC patients who desire fertility preservation, with no observed side effects of weight gain [49]. Metformin has demonstrated safety and good tolerability in FST, and its addition to MPA has been found to be more effective in treating obese patients [28]. In our previous study, we established that metabolic syndrome and its related factors are independent risk factors for CR, and incorporating metabolic syndrome into the prediction ROC curve significantly improves prediction accuracy [25, 50]. However, there is a lack of studies that integrate various risk factors and provide guidance for prognosis. Our study is the first to develop a comprehensive evaluation system for FST patients. The predictive nomogram constructed in this research exhibits high accuracy in predicting the prognosis of FST and can guide the treatment process.

The primary limitation of our study is its retrospective design conducted at a single center, where multiple conservative treatment regimens were employed for these patients. Additionally, some cases were lost to follow-up, and clinical factors were solely based on medical records, but not a prospective study. This study is based on a real world study, and the information we collected might not match in two groups. Furthermore, we enrolled all of the patients between the time period and a number of patients were still undergoing treatment at the time of the last follow-up, which may have influenced the research outcomes. In the future, we will include more patients with treatment outcomes, which can reduce bias. However, this study represents one of the largest comprehensive analyses of risk factors for CR in AEH or EEC patients.

## Conclusion

In order to further improve the prognosis of patients with FST, our study has developed a highly accurate predictive nomogram that incorporates multiple factors to predict CR in for AEH and EEC patients. This nomogram can serve as a valuable guide for patients undergoing fertility preservation treatment. Moreover, we encourage future investigations in other centers to validate these findings and contribute to prospective research on nomograms for FST in AEH and EEC patients.

### Supplementary Information

**Additional file 1: Figure S1.** Calibration curve of the nomogram.
