# Supplementary figures and images for: Prediction of complete regression in fertility-sparing patients with endometrial cancer and apical hyperplasia: the GLOBAL model in a large Chinese cohort
Source: J Transl Med. 2024 Feb 2;22:127. doi: 10.1186/s12967-023-04671-w (PMC10837883; doi:10.1186/s12967-023-04671-w)

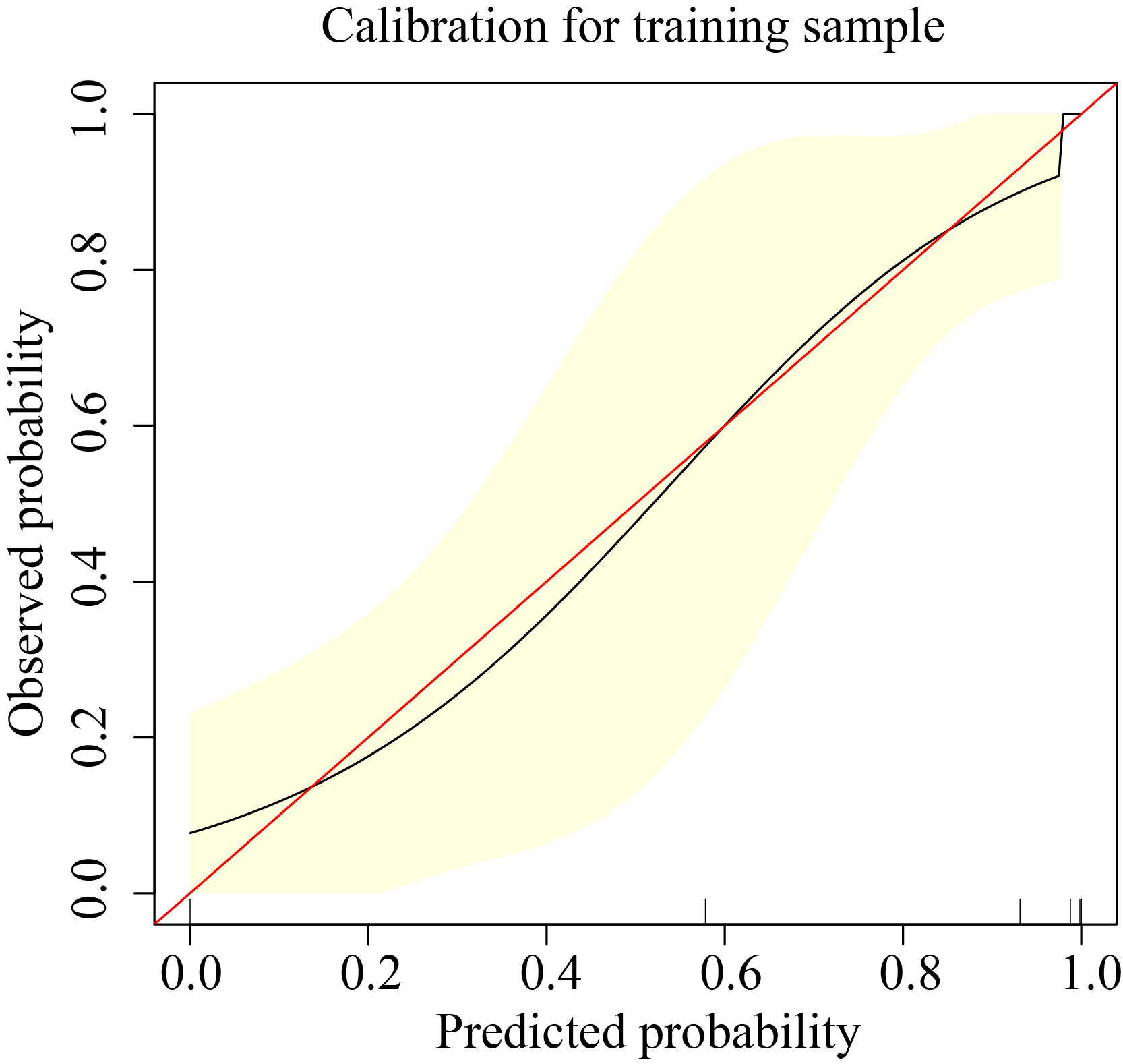

Supplement: Supplementary file 1 — Additional file 1: Figure S1. Calibration curve of the nomogram. [file 12967_2023_4671_MOESM1_ESM.jpg]
